# Identifying individuals at risk of developing psychosis: A systematic review of the literature in primary care services

**DOI:** 10.1111/eip.13365

**Published:** 2023-01-11

**Authors:** Jerica Radez, Felicity Waite, Emma Izon, Louise Johns

**Affiliations:** ^1^ Oxford Institute of Clinical Psychology Training and Research, Medical Sciences Division, University of Oxford Oxford UK; ^2^ Oxford Health NHS Foundation Trust Oxford UK; ^3^ Department of Psychiatry University of Oxford Oxford UK

**Keywords:** at‐risk mental state, general practitioner, primary care, screening

## Abstract

**Aim:**

Psychosis and related disorders are a major public health issue. Early identification and prevention for those at high risk (at‐risk‐mental‐state, ARMS) is important. General practitioners (GPs) are often the first point of contact for health services. In this review we aim to identify (1) the most common methods for identifying individuals with an ARMS in primary care, (2) the methods for improving identification of individuals with an ARMS in primary care, and (3) the most common barriers that prevent GPs from screening for individuals with an ARMS.

**Methods:**

We conducted a systematic review (PROSPERO 42021245095) of quantitative and qualitative studies with no date restriction. Searches were performed in September 2021. Studies' quality was appraised using Mixed Methods Appraisal tool (MMAT).

**Results:**

We identified 16 eligible studies, and all but one provided quantitative data. Nearly two‐thirds of studies were classified as ‘medium’ quality. Employing narrative synthesis, we identified three themes relating to (1) improving GP knowledge and confidence in identifying individuals with an ARMS, (2) balancing the over‐ and under‐identification of individuals with an ARMS in primary care, and (3) supporting GPs as significant stakeholders in early diagnosis and treatment of individuals with an ARMS.

**Conclusions:**

Improved identification of individuals with an ARMS is needed. We identified various strategies, including development and implementation of identification methods (e.g., screening measures), educational interventions for GPs (e.g., workshops), and systemic interventions (e.g., simplifying referrals to secondary care, developing integrated services). When implemented successfully, these interventions may help facilitate the access to appropriate care for individuals with an ARMS.

## 1 INTRODUCTION

Psychosis is defined as an abnormal mental state characterized by the presence of delusions, hallucinations, or both (APA, 2022). Psychosis and related disorders are a significant public health issue, especially due to the young age at onset, high levels of associated impairment and high prevalence of comorbid physical and mental health conditions (Anderson, 2019; Rössler et al., 2005). Preventive health strategies, such as identifying individuals at risk of developing psychosis, have the potential to prevent or delay the onset of a first psychotic episode (Fusar‐Poli et al., 2013) and to improve the outcomes of those who do later develop psychosis (Valmaggia et al., 2015). However, only a minority of individuals who develop psychosis are identified before development of psychosis, that is, whilst ‘at risk’ (McGorry et al., 2018). Early identification in everyday settings, such as in schools or primary care, could facilitate access to appropriate and timely treatment (Fusar‐Poli et al., 2020).

Over the past few decades, research has demonstrated that psychosis‐like experiences (PLEs), such as subclinical hallucinations and delusions, occur on a continuum, rather than the historic categorical conceptualisation. This dimension includes general public, those at high risk, and people experiencing psychosis (Bebbington et al., 2013; Unterrassner et al., 2017). When accompanied by decline in functioning, PLEs are one of the key markers of a clinical high‐risk for psychosis (Fusar‐Poli et al., 2013). The importance of this concept and its predictive values for psychosis and other mental health problems, including anxiety and depression, has been increasingly recognized, and therefore, many argue that it should be included in the main section of the DSM‐5 revision (Corcoran et al., 2021). However, unlike most other mental health problems in the DSM‐5, the concept of high‐risk for psychosis has been constantly evolving. More recent studies suggest that symptoms such as anxiety, low mood, and substance use are equally important as PLEs when identifying those at risk for developing psychosis (Carrión et al., 2013; Fusar‐Poli et al., 2013). The lack of a clinical consensus and overlapping symptoms between those at risk of developing psychosis and those experiencing other mental health problems make early identification of these individuals particularly tricky.

Several definitions have been used to describe individuals who are at risk of developing psychosis (Miller et al., 2003; Yung & McGorry, 1996). In this review we use the term ‘at‐risk mental state’ (ARMS) as set out by A. Yung et al. (1998). This framework defines three criteria that indicate being at‐risk: (1) experiencing attenuated psychotic symptoms (APS; sub‐threshold frequency or intensity), (2) brief limited intermittent psychotic symptoms (BLIPS; with these symptoms resolving spontaneously within a week), (3) genetic vulnerability to developing a psychotic disorder (Thompson et al., 2016). All three groups require a drop in functioning for at least 1 month within the previous 12 months. A person is considered ‘at risk’ if they meet one or more of these criteria. Second criterion is the one most associated with transition to psychosis (Fusar‐Poli et al., 2017). ARMS is most commonly identified in young people, aged 15–25, which is the period associated with the highest risk of developing first episode psychosis (Thompson et al., 2016).

There are three main routes for identifying individuals with an ARMS, including primary prevention (e.g., universal screening in schools), secondary prevention (e.g., screening those at risk in GP surgeries) and tertiary prevention (e.g., specialist mental health services) (Fusar‐Poli et al., 2019). Individuals with an ARMS are usually identified in specialized early intervention clinics (Howie et al., 2019). However, detection of individuals with an ARMS via specialized clinics misses a significant proportion of people who later develop psychosis, and therefore outreach campaigns, involving other community stakeholders (e.g., schools, GPs) are instrumental to improve early detection rates (Fusar‐Poli et al., 2019).

The first clinical and research clinic for detection and treatment of individuals with an ARMS in the world was The Personal Assessment and Crisis Evaluation – PACE Clinic in Australia (Yung et al., 1996), which worked closely with the GPs, schools, universities and other support agencies for young people (Yung et al., 2007). Many other countries, including the UK, Norway, Denmark, and Canada, have since developed similar ARMS clinics and (assertive) community outreach strategies aimed at early detection and treatment of individuals with an ARMS. These programmes have generally been well accepted by patients and their support networks (Jackson & McGorry, 2009). However, individuals identified within these services often represent a small percentage of those who will develop psychosis (e.g., Murguia‐Asensio et al., 2013) and therefore, further work with the main stakeholders and referrers needs to be done to enhance the early detection strategies (Power et al., 2007).

Primary care and educational practitioners have access to a wide range of young people and therefore these settings provide an opportunity for potential early identification of individuals with an ARMS (Kennedy et al., 2020). A recent systematic review of nine studies looking at identifying individuals with an ARMS in educational settings identified a number of screening tools used in schools (e.g., Prodromal Questionnaire – PQ), and a relatively large proportion (i.e., up to 40%) of individuals who scored above the ARMS threshold. This suggests that higher‐than‐recommended thresholds might be used to identify ARMS individuals more accurately in non‐clinical settings (Howie et al., 2019). Notably, one third of the studies also identified that young people with ARMS also had other comorbid problems (most commonly anxiety and depression), highlighting the complex clinical picture of these participants. Therefore, more sensitive measures assessing a whole range of symptoms might be more efficient than identifying individuals that only experience psychosis‐like symptoms (van Os & Guloksuz, 2017).

Similarly to schools, primary care practitioners (GPs) are particularly well placed to identify individuals with an ARMS as they often represent the first port of call for individuals with psychological problems and act as ‘gatekeepers’ between primary care and specialist mental health services (Strelchuk et al., 2021). Indeed, a recent systematic review of pathways to care (i.e., the time between symptom onset, first professional contact and the beginning of an appropriate treatment) in ARMS identified GPs to be one of the key pathway agents (Allan et al., 2020). In addition, each person – regardless of their background – can access a GP, and the average number of GP visits per person per year is around 4 in the United Kingdom (Hobbs et al., 2016). This enables GPs to identify individuals with an ARMS from various backgrounds, including those from Black and Minority Ethnic (BAME) backgrounds – individuals who are particularly under‐represented in specialist mental health services (Beck et al., 2019). However, the success of primary care practitioners in identifying individuals with an ARMS remains unclear. To date there has been no systematic assessment of identifying individuals with an ARMS in primary care.

In this systematic review we set out to understand the role of primary care practitioners in identifying individuals with an ARMS. There are three research questions:

1. What are the most common methods (e.g., screening tools, interviews) for identifying individuals with an ARMS in primary care?
2. What are the methods of improving identification of individuals with an ARMS in the primary care setting?
3. What are the most common barriers that prevent primary care practitioners from screening for ARMS?

## 2 METHOD

This systematic review followed the updated version of the Preferred Reporting Items for Systematic Reviews and Meta‐Analyses (PRISMA) statement (Moher et al., 2009; Page et al., 2021). A PRISMA checklist is provided in Supporting information S1. The review's protocol was registered with the International Prospective Register of Systematic reviews (PROSPERO) in April 2021 (registration number: CRD42021245095).

### 2.1 Literature search

The search terms captured three major concepts: (1) at‐risk mental state, (2) primary care, and (3) screening (see Supporting information S2 for details). To estimate the number of records and to inform the final search strategy we conducted scoping searches in February 2021. These searches identified approximately 2000 search results from multiple databases. As the search strategy was revised after conducting scoping searches, the identified 2000 records were not included in the final set of records. The final search was conducted in September 2021 using the NHS Evidence Healthcare database, which combines Medline, PsychINFO and Embase. In addition, we searched the Web of Science Core Collection. Hand‐searching methods were also used to check the reference lists of identified papers in the full text screening stage. We performed backward and forward reference searching for papers that met the eligibility criteria in the initial searches.

### 2.2 Eligibility criteria

The study was included if it reported (1) details about the methods (e.g., screening tools) for identifying individuals with an ARMS in primary care AND/OR (2) methods/interventions to improve the identification of individuals with an ARMS in primary care AND/OR (3) barriers for screening/identifying individuals with an ARMS in primary care. Studies that reported relevant data prospectively and/or retrospectively were included. We included qualitative as well as quantitative and mixed methods studies. In addition, the study was included if participants were recruited through primary care services (i.e., GPs or individuals accessing primary care services). Finally, the study was included if the manuscript was accessible in English and published in a peer review journal. Theoretical articles and systematic reviews/meta‐analyses on related topics, as well as studies only reporting pathways to care to ARMS services were not included.

### 2.3 Data extraction

Data extraction forms were developed within the research team and included the following information: (1) Area of focus (ARMS identification tools, barriers to identification, or strategies to improve identification), (2) methodology used (quantitative, qualitative, or mixed methods), (3) country, (4) number of participants, (5) participants' age, (6) percentage female participants, and (7) key findings in relation to the review's research question. Data extraction was led by JR, who extracted the data for all identified papers. Data from 50% (*n* = 8) of studies were also independently extracted by the second reviewer (EI). In case of discrepancies between the reviewers, a third member of the research team (LJ/FW) was consulted.

### 2.4 Quality rating

We used the Mixed Methods Appraisal Tool – MMAT (Hong et al., 2018). The MMAT was chosen due to the high heterogeneity of the studies. The MMAT permits the reviewer to appraise the quality of five categories of studies – qualitative, randomized controlled trials, non‐randomized studies, quantitative descriptive studies, and mixed methods studies (Hong et al., 2018). Further, the MMAT has favourable psychometric characteristics, with intra‐class correlations ranging from 0.7 to 0.9 indicating moderate to perfect agreement between different reviewers (e.g., Pace et al., 2012). JR assessed the quality of all included studies, and the second reviewer (EI) assessed the quality of 50% (*n* = 8) of the studies. Any discrepancies between the reviewers were discussed and resolved within the research team. Quality ratings (total scores) are reported in Table 1. Based on the total sum score, each study was classified as ‘low’ (total sum score ≤ 2), ‘medium’ (total sum score of 3 or 4) and ‘high’ (total sum score of 5). Individual item ratings for each study are reported in Supporting information S3.

**TABLE 1:** Characteristics of included studies

First author (year)	Study focus	Study aim	Study type (subtype)^a^	Number of participants	Country	Age	Females (%)	Study findings in relation to research question	Quality rating – Total
French et al. (2012)	ARMS identification tool (RQ1)	To assess the ability of the Primary Care Checklist (PCCL) to accurately identify individuals with an ARMS.	Quantitative (descriptive)	176 (83% met the diagnostic criteria for ARMS); 37 (21%) screened with PCCL by their GP	UK	14–34 (*M* = 20.78, SD = 4.16)	37.5% ^b^	*Instrument used*: PCCL checklist (French & Morrison, 2004) *Findings*: PCCL checklist has high sensitivity and low specificity in identifying ARMS adolescents. Better sensitivity/specificity ratio for short 6‐item version or the original version with different weighting	4 (medium)
Quijada et al. (2010)	ARMS identification tool (RQ1)	To describe and evaluate an ARMS screening programme and the demographic and clinical characteristics of people presenting to the service.	Quantitative (descriptive)	20^b^ individuals with an ARMS	Spain	14.7–16.8^b^	40%^b^	*Instrument used*: Spanish version of ERIraos checklist (Maurer et al., 2006) *Findings*: ERIraos checklist could help identifying individuals with an ARMS in primary care.	3 (medium)
Chen et al. (2019)	Strategy to improve identification of ARMS in primary care (RQ2)	To identify common symptoms and patterns of symptoms presented to the GPs prior to the diagnosis of first psychotic episode.	Quantitative (non‐RCT – Case–control study)	3045 patients with recorded FEP and 12 180 controls	UK	16–45 (Me = 30)	37.1%	*Strategy*: Examination of patients' medical records *Findings*: *Patterns of consultations*: Higher number of GP consultations in patients who later develop psychosis *Symptoms*: Mood disorders, ‘neurotic’ symptoms, behavioural change in volition, substance misuse, physical symptoms, perceptual changes (relatively rarely, but significantly more common than in healthy controls). Three distinct prodromal patterns: (1) no/minimal symptoms cluster (if symptoms, then mood or physical health), (2) Mood cluster (most commonly 2 symptoms, e.g., low mood and ‘neurotic’ symptoms), (3) multiple symptom cluster (e.g., mood, physical health, behavioural problems). The first two clusters were more common. Cluster one likely youngest and male; cluster three likely older and more likely female and long DUP. *Time consultation‐diagnosis*: 2–2.5 years (shorter for perceptual changes).	5 (high)
Falloon et al. (1996)	Strategy to improve identification of ARMS in primary care (RQ2)	Evaluation of the ‘Buckingham project’ – collaboration between GPs and specialist mental health services. (pilot study)	Quantitative (descriptive)	18 GPs	UK	n/a	n/a	*Strategy*: Different service set‐up *Findings*: Formal screening for ARMS in GP setting, combined with family and specialized mental health support resulted in reduced incidence of schizophrenia in the area.	Not assessed
Perez et al. (2015)	Strategy to improve identification of ARMS in primary care (RQ2)	Establishing if 1) low intensity (postal information campaign) or 2) high intensity (postal information + theory‐based educational intervention) lead to different outcomes in terms of the number of ARMS referrals from primary care.	Quantitative (RCT)	30 GP practices included in high‐intensity intervention and 34 in low intensity intervention (from Peterborough and Cambridgeshire)	UK	n/a	n/a	*Strategy*: ARMS educational intervention *Findings*: High intensity intervention was more effective than low intensity intervention in increasing the number of referrals to first episode psychosis and ARMS services. High number of true and false positives referred via the high intensity group. Intervention was costly but has a potential to lead to long‐term savings due to earlier detection/intervention. Low intensity intervention was no more efficient than no intervention.	5 (high)
Platz et al. (2006)	Strategy to improve identification of ARMS in primary care (RQ2)	To assess help‐seeking behaviours and main presenting symptoms of individuals with an ARMS presenting to the GPs.	Quantitative (non‐RCT – Cohort study)	50 individuals with an ARMS	Switzerland	21	38%	*Strategy*: Examination of patients' self‐reported symptoms and help‐seeking behaviour *Findings*: *Symptoms*: Depression, social decline, social withdrawal. ‘Typical’ psychosis symptoms (e.g., hallucinations) were less common/rare compared to the first‐episode psychosis group. *Patterns of consultations*: GPs were most consulted for negative/non‐psychosis‐specific symptoms (e.g., depression).	3 (medium)
Reynolds et al. (2015)	Strategy to improve identification of ARMS in primary care (RQ2)	Evaluation of GP training (1 session) on ARMS recognition and referrals to appropriate service.	Quantitative (non‐RCT – Cohort study)	29 GP practices; 54 individuals referred/identified as ARMS by the GPs	UK	*M* = 21.85 (SD = 4.16)	41%	*Strategy*: ARMS educational intervention *Findings*: 50% of referrals by the GPs were correctly identified as ARMS. 1 h GP training increased the number of ARMS (but not EIP) direct referrals. Increased number of false and true positives.	3 (medium)
Simon et al. (2010)	Strategy to improve identification of ARMS in primary care (RQ2)	To see if a repeated exposure (sensitisation) to the clinical vignette can improve diagnostic knowledge of ARMS in GPs.	Quantitative (non‐RCT – Cohort study)	750 GPs^b^	Switzerland	n/a	n/a	*Strategy*: ARMS educational intervention *Findings*: GPs exposed to the intervention showed a significant improvement in diagnostic knowledge of ARMS at 6‐ and 12‐month follow‐up (the effect persisted after sensitisation). This was not observed for non‐sensitized GPs. Sensitized GPs also improved their knowledge of symptoms of ARMS that are often under‐identified (e.g., social withdrawal and functional decline).	3 (medium)
Sullivan et al. (2018)	Strategy to improve identification of ARMS in primary care (RQ2)	To see if a primary care consultation pattern for ARMS can be used to identify patients who later develop psychosis.	Quantitative (non‐RCT – Case–control study)	530 primary care practices; 11 690 patients with psychosis and 81 793 matched controls	UK	*M* = 51.34 (SD = 21.75)	57.4% ^b^	*Strategy*: Examination of patients' medical records *Findings*: *Symptoms*: Bizarre behaviour, suicidal behaviour (strongest predictor), cannabis‐associated problems, depressive symptoms, blunted affect, ADHD‐like symptoms, OCD‐like symptoms, social isolation, role functioning problems, mania symptoms, sleep disturbance, smoking‐associated problems. Positive predictive value of symptoms greater for men than women. *Patterns of consultations*: More common GP consultations; increasing number of consultations over time.	5 (high)
Jacobs et al. (2011)	Barriers/facilitators to identifying ARMS in primary care (RQ3)	Understanding GPs appraisals of ARMS.	Quantitative (descriptive)	72 GPs	US	*M* = 52.7 ^b^ (Me = 53.0)	47.6% ^b^	*Barriers*: Lack of knowledge about ARMS (i.e., thinking about it as schizophrenia spectrum); lack of diagnostic category to capture the symptoms of ARMS.	3 (medium)
Jacobs et al. (2012)	Barriers/facilitators to identifying ARMS in primary care (RQ3)	Exploring how different practitioners (GPs, clinical psychologists and psychiatrists) treat individuals with an ARMS.	Quantitative (descriptive)	68 primary care practitioners	US	*M* = 52.6 (SD = 10.9)	48% ^b^	*Barriers*: Lack of knowledge about ARMS, its identification and treatment.	3 (medium)
Russo et al. (2012)	Barriers/facilitators to identifying ARMS in primary care (RQ3)	To identify factors that influence the identification of individuals with an ARMS in primary care using theory of planned behaviour.	Quantitative (descriptive)	82 GPs	UK	*M* = 45.6 (SD = 9.4)	47%	*Barriers*: Thinking that their peers (e.g., psychiatrist) would not approve them diagnosing individuals with an ARMS (systemic barriers); low level of confidence and perceived control over identification of ARMS, lack of skills. *Facilitators*: Positive attitudes and intentions towards identifying individuals with an ARMS, high personal motivation/interest in ARMS and mental health, knowledge of patient and their background.	3 (medium)
Simon et al. (2009)	Barriers/facilitators to identifying ARMS in primary care (RQ3)	The international GP study on early psychosis ‐ to assess the diagnostic knowledge, treatment practices, attitudes, and preferences for support of GPs in different countries.	Quantitative (descriptive)	2784 GPs	International ‐ Switzerland, Austria, UK, New Zealand, Czech Republic, Canada, Australia, Norway	*M* = 46.4 (SD = 9.44)	45.30%	*Barriers*: Lack of knowledge about ARMS (about symptoms of ‘functional decline’), lack of low‐threshold services to refer individuals with an ARMS to. *Facilitators*: Good knowledge of ‘positive symptoms’ of psychosis; being a ‘gatekeeping GP’ (have better diagnostic knowledge than non‐gatekeeping GPs), practicing in ‘Commonwealth’ countries (have better diagnostic knowledge than continental European GPs).	3 (medium)
Smith et al. (2021)	Barriers/facilitators to identifying ARMS in primary care (RQ3)	To understand GPs' comfort and understanding of ARMS; to understand GPs' interest in specialized training.	Quantitative (Descriptive)	75 GPs	Australia	n/a	n/a	*Barriers*: Lack of knowledge about ARMS (31% of GPs not aware of the concept of ARMS) *Facilitators*: Motivation for further training (almost all (95%) of GPs interested in further training in YP mental health).	3 (medium)
Strelchuk et al. (2021)	Barriers/facilitators to identifying ARMS in primary care (RQ3)	To investigate GPs views about identifying individuals with an ARMS in primary care. To identify barriers and facilitators related to the identification.	Qualitative	20 GPs	UK	32–63 (*M* = 46.0, SD = 8.6)	40%	*Barriers*: Lack of knowledge about ARMS, lack of mental health training, diagnostic similarities between ARMS and other mental health problems, lack of diagnostic categories (e.g., ARMS), lack of time (short GP consultations), difficulties making appointment with the GP, high threshold for accessing secondary care, difficulties about getting an appointment in secondary care, fears about labelling patients, patients not seeking help due to lack of motivation, depression and stigma, *Facilitators*: Increasing knowledge about specialist referrals and ARMS treatment.	5 (high)
Tor and Lee (2009)	Barriers/facilitators to identifying ARMS in primary care (RQ3)	To compare attitudes of Singapore psychiatrists vs. GPs about ARMS.	Quantitative (descriptive)	107 primary care practitioners	Singapore	57.9% aged between 30 and 40	51.40%	*Barriers*: Lack of knowledge about ARMS (GPs more likely to diagnose patients with psychosis), lack of confidence in identifying ARMS (less than a third of GPs advocate for screening for ARMS in high‐risk groups), low confidence in treating individuals with an ARMS (almost all GPs not wanting to treat ARMS), low tolerance of psychosis‐like symptoms.	2 (low)

Abbreviations: ADHD, attention‐deficit hyperactivity disorder; EIP, early intervention in psychosis; ERIraos, Interview for the Retrospective Assessment of the Onset and Course of Schizophrenia and Other Psychoses (German version); n/a, the study did not report participants' gender or age; OCD, obsessive–compulsive disorder; RQ1‐3, Research Question 1–3, PCCL, Primary Care Checklist; YP, young people.

^a^ Study type as defined by MMAT (quality appraisal tool).

^b^ Study characteristics reported in relation to the whole sample (i.e., sub‐group statistics were not reported).

### 2.5 Data synthesis

Data were analysed using narrative synthesis and following ESRC guidance (Popay et al., 2006). Narrative synthesis is a method of data analysis for systematic reviews including a wide range of study formats (e.g., qualitative, quantitative, mixed methods) that might otherwise make statistical approaches less feasible (Barnett‐Page & Thomas, 2009). We followed synthesis without meta‐analysis (SWiM) reporting guidance (Campbell et al., 2020) to ensure that narrative synthesis was conducted according to the ESRC guidance.

Data synthesis began with preliminary synthesis (Popay et al., 2006). This included creating short textual descriptions of studies (i.e., producing a descriptive paragraph for each study), which enabled the reviewers to become familiarized with each study. The following step included tabulation of studies according to their (1) methodology used, (2) study aims, (3) participant group (i.e., GPs or individuals with an ARMS), (4) participant gender, (5) participant age, (6) study results, and (7) implications. Information regarding each study's quality appraisal was also included. This was followed by creating a ‘common rubric’ (common framework) – organizing the results of all studies in a meaningful way and in relation to the review's aims. For instance, the common rubric for research question 1 included the details about the screening tool used (e.g., screening questionnaire) and main study findings in relation to the utility of the screening tool in primary care setting. The final stage of preliminary synthesis was a Thematic Analysis. Information extracted in ‘common rubrics’ was treated as codes, which were then grouped and organized in an inductive manner (i.e., without being driven by a set of a priory themes/review's aims). For instance, codes ‘sensitivity/specificity issues’ and ‘false positives’ were combined in a family of codes/subtheme called ‘identification issues’ which then formed a significant part of the main theme (Theme 2). The final set of themes was generated analytically – providing the interpretation ‘beyond’ the primary review's aims and generating a story about the review's findings (e.g., Thomas & Harden, 2008).

The third step of analysis included exploring the relationship within the studies. For instance, we explored the relationship within a group of studies with the same research question, which included comparison of their findings and exploring the relationship between these studies and identified themes. We also explored the relationships between the studies, which included comparing studies conducted via different methodologies and in different countries. We further explored variability in outcomes, designs, and populations of included studies, and investigated whether this variability affects our main themes identified in the previous step of Narrative Synthesis. Any identified pattern of difference was reported in Section 3.

In the final step of narrative synthesis (assessing robustness) we assessed the robustness of the synthesis by removing the studies with the lowest quality and investigating whether this affected the results.

## 3 RESULTS

### 3.1 Study selection

**FIGURE 1:**
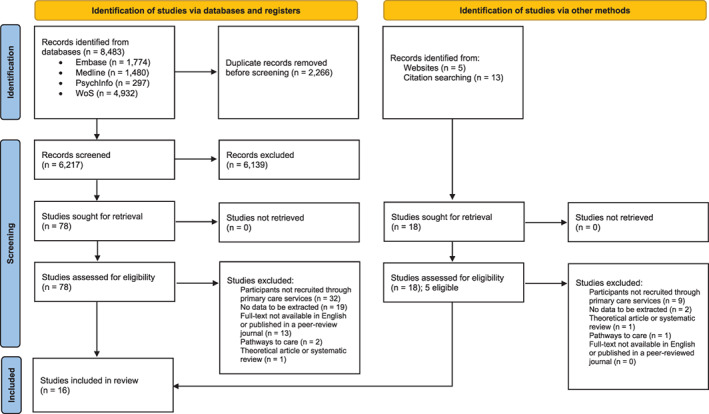
PRISMA flowchart of study selection process

In total, 8430 records were identified from databases. After duplicates were removed, 6217 abstracts and 96 full texts were screened. Sixteen studies were identified as eligible and are included in the current review. Study selection was led by the first author (JR), who screened all abstracts and full texts. A proportion of records (20% of abstracts and 25% of full texts) were independently screened by a second reviewer (EI) and the agreement between the reviewers was very good (*κ* > .81). The full process of study selection is presented in the PRISMA flowchart (Figure 1).

### 3.2 Study description

Sixteen studies were identified, with 15 studies providing quantitative and one study providing qualitative data. Two (12.5%) studies were exploring methods of identifying individuals with an ARMS in primary care, seven (43.8%) studies explored ways of improving the identification of ARMS in primary care, and the remaining seven (43.8%) studies examined barriers to identifying individuals with an ARMS in primary care. Study characteristics are presented in Table 1. Studies are ordered by research question and alphabetically within that.

In the majority (75.0%) of the studies, participants were GPs, whilst in the remaining 25.0% of studies, participants were individuals with an ARMS or first episode psychosis (FEP) patients. Studies varied considerably in terms of country (with 50.0% of studies conducted in the United Kingdom, 12.5% in the United States, 12.5% in Switzerland, 6.3% in Australia, 6.3% in Spain, 6.3% in Singapore and 6.3% across multiple countries); age range in years (from 32 to 63^1^ for GP participants and from 14 to 45 for patient samples); sample size (from 18 to 2784 for GP participants and from 20 to 3045 for patient samples); gender (percentage of females ranging from 40.0% to 57.4% for GP participants, and from 37.1. to 40.0% for patient samples). Notably, several (25.0%) studies did not report participant age and the same proportion of studies did not report participant gender.

### 3.3 Quality ratings

Studies assessed varied considerably in terms of quality. The majority of studies (62.5%) were classified as ‘medium’ quality, a quarter of the studies were classified as ‘high’ quality and one study (6.3%) as ‘low’ quality. One of the included studies did not meet the MMAT criteria for quality appraisal (i.e., the study did not pass two screening questions for quality appraisal); however, the study was still included in the review. Strengths of the studies usually included appropriate sampling strategies, data analysis methods, and descriptions of measures. Limitations of the studies usually related to absence of detail regarding sample representativeness and lack of information on risk of no response bias.

### 3.4 Identified themes

We identified three themes that were common across all included studies. The themes were named (i) Improving GP knowledge and confidence in identifying individuals with an ARMS, (ii) Balancing over‐ and under‐identification of individuals with an ARMS in primary care, and (iii) Supporting GPs as significant stakeholders in early diagnosis and management of individuals with an ARMS. The relationship between the themes and this review's aims is outlined in Table 2 and the content of each theme is summarized below.

**TABLE 2:** Identified themes in relation to the review's aims

Aim	Results	Theme
Aim 1: The most common methods (e.g., screening tools, interviews) for identifying ARMS in primary care	Two tools identified: PCCL checklist (French & Morrison, 2004) ERIraos checklist (Maurer et al., 2006)	Theme 2: Balancing over‐ and under‐identification of individuals with an ARMS in primary care
Aim 2: Methods of improving identification of ARMS in primary care	Educational interventions for GPs Optimizing cut‐off values of existing tools Using medical‐record‐based prognostic models	Theme 2: Balancing over‐ and under‐identification of individuals with an ARMS in primary care
	Providing specialist input within primary care practices	Theme 3: Supporting GPs as significant stakeholders in early diagnosis and treatment of individuals with an ARMS
Aim 3: The most common barriers to screening for ARMS in primary care	Lack of knowledge about ARMS Lack of confidence in treating ARMS	Theme 1: Improving GP knowledge and confidence in identifying individuals with an ARMS
	Limited time for individual consultations High threshold for secondary care mental health services Long waiting times Patient‐experienced stigma	Theme 3: Supporting GPs as significant stakeholders in early diagnosis and treatment of individuals with an ARMS

#### 3.4.1 Theme 1: Improving GP knowledge and confidence in identifying individuals with an ARMS

The majority of studies identified a lack of knowledge of ARMS amongst GPs (Jacobs et al., 2011; Jacobs et al., 2012; Russo et al., 2012; Simon et al., 2009; Smith et al., 2021; Strelchuk et al., 2021; Tor & Lee, 2009). Furthermore, GPs reported not feeling confident about treating individuals with an ARMS (Jacobs et al., 2011; Tor & Lee, 2009) and finding it hard to distinguish ARMS from other common mental health disorders due to a lack of a single diagnostic category and overlap between ARMS and other mental health problems (Jacobs et al., 2011; Strelchuk et al., 2021). GPs seem to be more aware of ‘positive’ ARMS symptoms (e.g., hallucinations), rather than symptoms of functional decline (Simon et al., 2009). This is important, as studies suggest that individuals with an ARMS most commonly consult their GPs for non‐psychosis‐specific symptoms (e.g., depression, social withdrawal, obsessive–compulsive disorder‐like symptoms) (Chen et al., 2019; Platz et al., 2006; Sullivan et al., 2018). Notably, some studies (e.g., Sullivan et al., 2018) suggest that GPs should be particularly mindful when identifying the non‐psychosis‐specific symptoms in young men since these symptoms seem to be a particularly strong predictors of ARMS in this population.

#### 3.4.2 Theme 2: Balancing over‐ and under‐identification of individuals with an ARMS in primary care

Simple (e.g., single session) interventions can improve GPs knowledge of ARMS (Simon et al., 2010) and improve GPs' identification of individuals with an ARMS in primary care (Perez et al., 2015; Reynolds et al., 2015). Similarly, clinician‐administered ARMS screening checklists, such as the Early Detection Primary Care Checklist – PCCL (French & Morrison, 2004) and The Early Recognition Inventory – ERIraos (Maurer et al., 2006), can potentially help with early identification of individuals with an ARMS in primary care (French et al., 2012; Quijada et al., 2010). However, although GPs seem to be interested in receiving further training on identifying individuals with an ARMS (Smith et al., 2021), research suggests that educational interventions and an ARMS checklist can often lead to a large number of false positives (French et al., 2012; Perez et al., 2015; Reynolds et al., 2015). Modifying the scoring criteria of existing checklists (French et al., 2012), using tailored cost‐effective interventions (Perez et al., 2015; Reynolds et al., 2015), and developing a medical‐record‐based prognostic model of identifying individuals with an ARMS in primary care (Sullivan et al., 2018) all have the potential to outweigh the benefits of over‐identifying individuals with an ARMS in primary care.

#### 3.4.3 Theme 3: Supporting GPs as significant stakeholders in early diagnosis and treatment of individuals with an ARMS

GPs are often familiar with their patients, so they are well placed for identifying individuals with an ARMS (Russo et al., 2012). However, some logistical barriers, such as limited time for individual consultations, high threshold for secondary care mental health services, and long waiting times, represent important obstacles for identifying individuals with an ARMS in primary care (Simon et al., 2009; Strelchuk et al., 2021). GPs also reported concerns about patient‐experienced stigma related to identifying individuals with an ARMS in their practices (Strelchuk et al., 2021), and concerns about other colleagues (e.g., psychiatrists) having doubts about GPs' abilities to accurately identify ARMS (Russo et al., 2012). Providing specialist input within primary care practices (i.e., integrated services) has the potential to improve GPs' abilities to confidently identify individuals with an ARMS in primary care (Falloon et al., 1996; Simon et al., 2009).

### 3.5 Robustness of the synthesis

To assess the robustness of the synthesis, we removed two studies – the study with the lowest quality rating and the study that did not meet the criteria for a quality appraisal (Falloon et al., 1996; Tor & Lee, 2009) and re‐examined the findings in relation to the identified themes. The main study findings and themes remained the same after excluding these studies.

## 4 DISCUSSION

### 4.1 Main results

This study identified and reviewed 16 studies addressing: (1) existing methods, (2) strategies to improve, or (3) barriers that prevent primary care practitioners from screening for ARMS in primary care. We identified three themes relating to GPs' knowledge and confidence in identifying ARMS in primary care, balancing the costs and benefits of identifying ARMS in primary care, and supporting GPs in early diagnosis/treatment of individuals with an ARMS.

GPs' knowledge about and confidence in identifying ARMS is generally low. Indeed, the findings suggest that GPs are well equipped for identifying PLEs, however, they often overlook the symptoms that are most strongly associated with ARMS, such as low mood, social withdrawal, and reduced functioning. Some strategies of improving GPs' knowledge of ARMS and early identification in primary care could include the use of screening tools (e.g., PCCL; French & Morrison, 2004), reviewing patients' medical records, and attending educational workshops on ARMS identification and treatment. Research suggests that these strategies are associated with higher proportions of correctly identified individuals with an ARMS in primary care. However, they also lead to a high proportion of false positives, which can be problematic, especially given the stigma associated with psychosis and related disorders (e.g., Strelchuk et al., 2021). Providing support for GPs on a systemic level (e.g., integrated services, such as OASIS in London) and developing screening tools that focus on a wide range of symptoms associated with ARMS (e.g., anxiety, low mood, social withdrawal) may lead to higher rates of correct identification of individuals with an ARMS (Fusar‐Poli et al., 2013).

The results of this review are broadly consistent with the existing literature. Problems with high rates of false positives and suboptimal sensitivity/specificity ratios of ARMS screening tools have been reported in a systematic review of ARMS screening tools in educational settings (Howie et al., 2019). Similarly, previous research also identified symptoms of affective disorders, reduced neurocognitive performance, functional impairments and non‐positive attenuated symptoms (e.g., motor disturbances) to be highly predictive of ARMS (Carrión et al., 2013; Howie et al., 2019), indicating that understanding of ARMS as a concept should be broad and not limited only to psychosis‐like symptoms. Previous studies have also demonstrated that using statistical modelling of patients' medical health records to improve the identification of certain mental health problems (most commonly using ‘deep learning’ – a form of artificial intelligence) has been effective in identifying mental health problems (Pham et al., 2017; Su et al., 2020). However, it is important to be aware of ethical implications of such prediction models as they can undermine patients' and clinicians' sense of agency, and shared decision making (Lane & Broome, 2022). Finally, previous research also identified systemic barriers related to the early identification of mental health problems in primary care, such as limited consultation time and long waiting times for specialist services (e.g., O'Brien et al., 2016), indicating the need for systemic changes in primary care. Expansion of primary‐care‐based mental health services, such as involvement of mental health professionals in decision making in primary care and integrated medical‐behavioural health care models, have both been associated with an increase identification of mental health problems in primary care (Asarnow et al., 2015; Haavet et al., 2021; Simon et al., 2009) and therefore, it is likely they could help GPs overcome barriers associated with identification of ARMS. However, only with the appropriate systemic changes, can we expect that interventions focused only on the GPs (e.g., educational workshops on ARMS) will be truly successful (Gask, 2007).

Our review identified several possibilities for further research. Firstly, there appears to be a lack of ARMS screening tools for use in primary care, and therefore, future research could focus on developing and validating short and easy‐to‐use ARMS screening questionnaires. Previous research with screening questionnaires for young people demonstrated that symptom impact questions often have a higher predictive value than disorder symptoms themselves (Evans et al., 2017; Goodman, 2001; Radez et al., 2021), and therefore, ARMS screening tools might achieve the optimal sensitivity/specificity ratios if including symptom impact questions. The short version of the Prodromal Questionnaire (PQ‐16) (Ising et al., 2012) is an example of a short self‐reported questionnaire for ARMS that includes the symptom impact items, and future research could investigate its utilization in primary care. In addition, future research should focus in identifying optimal cut‐off values for the ARMS questionnaires identified in current review (e.g., PCCL) for different populations (e.g., adults). Given a rapid expansion of the role of machine learning in mental health, future studies could also focus on further development and implementation of prediction models for identification of individuals with an ARMS based on their medical records and consultation patterns. Finally, we identified only one study that used qualitative in‐depth methodology to understand GPs' views about identifying and managing ARMS in primary care, and therefore, future qualitative research should further explore how GPs want to be supported when identifying and treating individuals with an ARMS in their practices.

### 4.2 Implications

This review's findings have clear practical implications. Firstly, there is a need to improve GPs knowledge and confidence in identifying individuals with an ARMS in primary care. Developing and validating quick and easy‐to‐use screening tools and software programs could help GPs identify individuals with an ARMS. Simple (i.e., one session) educational interventions could also aid early identification of ARMS in primary care. Educational interventions should also focus on educating GPs around potential barriers to (over)identification of individuals with an ARMS, such as misdiagnosis and unnecessary labelling of young people. Systemic factors, such as time for each GP consultation and difficulties making a referral to secondary care, need to be carefully considered when implementing identification of ARMS in primary care. Similarly, working closely with other community stakeholders and specialist mental health teams will likely make the above interventions more effective. Further, involving community stakeholders could also help GPs focus on other areas of psychosis prevention, such as reducing the exposure to risk factors (e.g., high potency cannabis use, see Murray et al., 2021) in those who may be at‐risk. Finally, our review identified that a lack of a clear diagnostic category for ARMS and use of multiple terms to describe individuals with an ARMS creates further confusion and reduces clinicians' confidence in identifying ARMS. Therefore, using the same name/diagnostic label could be beneficial.

### 4.3 Limitations

There are limitations to this review. Due to the high heterogeneity of included studies and reporting methods, it was not possible to conduct a meta‐analysis of ARMS screening tools or effectiveness of interventions to improve ARMS identification. Similarly, we were not able to compare study findings quantitatively in relation to the study characteristics (e.g., methodology used, country). Although we used broad search terms, which resulted in a high number of identified abstracts, a significant proportion of papers were identified using other (non‐database) searches (e.g., forward/backwards citation searching). This may be related to the nature of the ARMS concept and a wide range of definitions. It is important to acknowledge that none of the included studies investigated how rates of individuals with an ARMS identified via primary care compare to the rates of individuals with an ARMS identified via other settings (e.g., emergency departments, educational settings) and this question remains to be explored. Finally, only one study explored cross‐cultural differences (Simon et al., 2009) and therefore, future research could explore this further – in particular as ARMS service models vary significantly between different countries.

## 5 CONCLUSIONS

Early identification of those at high risk of psychosis has the potential to prevent or delay the onset of a first episode psychosis, with benefits for the individual, their families, as well as wider society. GPs are particularly well placed to identify individuals with an ARMS; however, as self‐identified by the GP participants included in this review, they often lack the appropriate knowledge and tools to do so. There are a number of interventions that could support GPs to identify individuals with an ARMS accurately and promptly, including developing and validating ARMS screening tools, delivering educational workshops for GPs, using machine learning to identify individuals with an ARMS based on their medical record patterns, simplifying referrals to secondary care services, and developing integrated services. Future development and implementation of these interventions may help individuals with an ARMS to access help promptly, and delay or even prevent the onset of psychosis.

## FUNDING INFORMATION

JR and EI were supported by funding for professional clinical psychology training by Oxford Health NHS Foundation Trust. Felicity Waite is funded by a Wellcome Trust Clinical Doctoral Fellowship (102176/B/13/Z).

## CONFLICT OF INTEREST

The authors declare that they have no conflict of interest.

## Supporting information


**DATA S1.** PRISMA checklist.

**DATA S2.** Search terms.

**DATA S3.** Quality ratings for each individual study.

## Data Availability

Data sharing is not applicable to this article as no new data were created or analyzed in this study.

## ENDNOTE

^1^
Estimated age range based on reported age ranges of GPs by 7 out of 11 studies.
