# Supplementary material for: Identifying individuals at risk of developing psychosis: A systematic review of the literature in primary care services
Source: Early Interv Psychiatry. 2023 Jan 11;17(5):429–46. doi: 10.1111/eip.13365 (PMC10946574; doi:10.1111/eip.13365)
Supplement: Supplementary file 2 — DATA S2. Search terms. [file EIP-17-429-s001.docx]

| At-risk mental state | Primary care | Screening |
| --- | --- | --- |
| ARMS | GP | Screen* |
| Ultra-high risk | G.P. | Detect* |
| Clinical high risk | General Practi* | Assess* |
| Brief limited intermittent psychotic symptoms | Family Practi* | Self* report |
| BLIPS | Primary Care | Question* |
| Attenuated psycho* symptom* | Primary Health Care | Identif* |
| Attenuated psycho* syndrome | Primary Care Physician* | Diagnos* |
| Basic symptom* | Family Physician* | Interview* |
| Prodrom* |  |  |
| Psycho* prodrom* |  |  |
| Gen* risk psychosis |  |  |
| Prepsychotic |  |  |

**Supporting information 2 – Search Terms**

**Table 1**

*Search terms*

(“At risk mental state” OR ARMS OR “Ultra-high risk” OR “Clinical high risk” OR “Brief limited intermittent psychotic symptoms” OR BLIPS OR “Attenuated psycho* symptom*” OR “Attenuated psycho* syndrome” OR “Basic symptom*” OR “Schizophreni* Prodrom*” OR “Psycho* prodrom*” OR “Gen* risk psychosis” OR Prepsychotic) AND (GP OR “G.P.” OR “General Practi*” OR “Family Practi*” OR “Primary Care” OR “Primary Health Care” OR “Primary Care Physician”* OR “Family Physician”* OR “Family Doctor*”) AND (Screen* OR Detect* OR Assess* OR “Self* report” OR Question* OR Identif* OR Diagnos* OR Interview*)
