# Supplementary material for: Identifying individuals at risk of developing psychosis: A systematic review of the literature in primary care services
Source: Early Interv Psychiatry. 2023 Jan 11;17(5):429–46. doi: 10.1111/eip.13365 (PMC10946574; doi:10.1111/eip.13365)
Supplement: Supplementary file 3 — DATA S3. Quality ratings for each individual study. [file EIP-17-429-s002.docx]

|  | Research questions (RQ) clear? | Collected data allow to address the RQs? | MMAT1 | MMAT2 | MMAT3 | MMAT4 | MMAT5 | Total^a^ |
| --- | --- | --- | --- | --- | --- | --- | --- | --- |
| French et al. (2012) | yes | yes | 1 | 0 | 1 | 1 | 1 | 4 |
| Quijada et al. (2010) | yes | yes | 1 | 1 | 0 | 1 | 0 | 3 |
| Chen et al. (2019) | yes | yes | 1 | 1 | 1 | 1 | 1 | 5 |
| Falloon et al. (1996) | no | no | Not applicable - treated as non-empirical research | | | | |  |
| Perez et al. (2015) | yes | yes | 1 | 1 | 1 | 1 | 1 | 5 |
| Platz et al. (2006) | yes | yes | 1 | 0 | 1 | 0 | 1 | 3 |
| Reynolds et al. (2015) | yes | yes | 1 | 1 | 1 | 0 | 0 | 3 |
| Simon et al. (2010) | yes | yes | 1 | 1 | 0 | 0 | 1 | 3 |
| Sullivan et al. (2018) | yes | yes | 1 | 1 | 1 | 1 | 1 | 5 |
| Jacobs et al. (2011) | yes | yes | 1 | 0 | 1 | 0 | 1 | 3 |
| Jacobs et al. (2012) | yes | yes | 1 | 0 | 1 | 0 | 1 | 3 |
| Russo et al. (2012) | yes | yes | 1 | 0 | 1 | 0 | 1 | 3 |
| Simon et al. (2009) | yes | yes | 1 | 0 | 1 | 0 | 1 | 3 |
| Smith et al. (2021) | yes | yes | 1 | 1 | 0 | 0 | 1 | 3 |
| Strelchuk et al. (2021) | yes | yes | 1 | 1 | 1 | 1 | 1 | 5 |
| Tor and Lee (2009) | yes | yes | 1 | 1 | 0 | 0 | 0 | 2 |

**Supporting Information 3 – Quality ratings for each individual study**

**Table 1**

*Quality ratings for each individual study*

^a^ Total sum score ≤ 2 = ‘low’, Total sum score of 3 and 4 = ‘medium’, Total sum score of 5 = ‘high’ quality.

MMAT1-MMAT5, 5 items assessing studies’ quality based on the Mixed Methods Appraisal Tool (MMAT, 2018, see http://mixedmethodsappraisaltoolpublic.pbworks.com/w/file/fetch/127916259/MMAT_2018_criteria-manual_2018-08-01_ENG.pdf )
